# Spontaneous Hemothorax in a Patient With von Recklinghausen’s Disease

**DOI:** 10.14740/jocmr1692w

**Published:** 2014-02-06

**Authors:** Marcel Rodriguez-Guzman, Belen Gallegos-Carrera, Sara Vicente-Antunes, Itziar Fernandez-Ormaechea, Jose Zapatero-Gaviria, Felipe Villar-Alvarez

**Affiliations:** a Department of Pneumology, IIS-Fundacion Jimenez Diaz, CIBERES, Madrid, Spain; b Department of Thoracic Surgery, IIS-Fundacion Jimenez Diaz, Madrid, Spain

**Keywords:** Spontaneous hemothorax, von Recklinghausen’s disease, Endovascular embolization, Arteriography

## Abstract

Type I neurofibromatosis (NF-1) is a rare autosomal dominant disease. It can affect any organ system including vascular tissues. A 53 years old man, with a past medical history of NF-1, retinitis pigmentosa and hypertension attended to the emergency department for chest pain and palpitations and was discharged 2 days after acute coronary syndrome was ruled out. During this admission an echocardiogram was performed which showed a left ventricular hypertrophy with normal ejection fraction and a chest X-ray which revealed no pathologic images. No invasive procedures were preformed. Three days after discharge, he returned to our hospital for sudden onset of oppressive chest pain in the right arm, irradiated to the ipsilateral shoulder, chest and back. After several tests, a diagnosis of hemothorax was made. Hemoglobin levels declined during the first 2 days of admission from 12.1 to 9.6 g/dL, although the patient remained hemodynamic stable. An arteriography was performed, which showed the presence of bleeding from a branch of the right subclavian artery, which was selectively catheterized and embolized with coils. Afterwards, a video-assisted thoracoscopy was made, in order to drain the hemothorax and to carry out a visual review of the pleural cavity. The patient had a good clinical and radiologic progression and was discharged after few days. After a year of follow-up, the patient has remained clinically asymptomatic with no further episodes of active bleeding.

## Introduction

Type I neurofibromatosis (NF-1) or von Recklinghausen’s disease is an autosomal dominant disease with an occurrence rate of 1 in 3,000 [1]. Its prevalence is similar in all races and sexes, and is usually diagnosed in adulthood, since its clinical characteristics develop throughout life. This entity can affect any organ system, especially connective, nervous and vascular tissues, and is characterized by skin tumors (neuromas) and abnormal cutaneous pigmentation [2].

Hemothorax is, by definition, a pleural fluid with a hematocrit greater than 50%. Most cases are related to trauma or invasive procedures such as thoracentesis, pleural biopsy or catheterization. Spontaneous hemothorax is rare, occurring in 3-7% of cases, and usually the causes include sneoplasm, vascular rupture, endometriosis or hematologic abnormalities such as hemophilia [3].

## Case Report

A 53 years old man, previously diagnosed with NF-1, retinitis pigmentosa, hypertension and following a treatment with atenolol, ramipril and NSAIDs, was admitted to our hospital for chest pain and palpitations, probably related to supraventricular tachycardia, and was discharged 2 days after being evaluated for acute coronary syndrome. During this admission an echocardiogram was performed which showed a left ventricular hypertrophy with normal ejection fraction and a chest X-ray which revealed no pathologic images. No invasive procedures were preformed.

Three days after discharge, he returned to our hospital with oppressive chest pain that began suddenly in the right arm, which then irradiated to the ipsilateral shoulder, chest and finally to the back. It was accompanied by dyspnea, profuse sweating and nausea, with one episode of vomiting. Except for occasional mild dysuria, there were no other accompanying symptoms. On physical examination, the patient was conscious, with adequate coloration of skin and mucous membranes and eupneic at rest. It caught our attention that during the cardiopulmonary auscultation, we detected decreased lung sounds, moderate to severe in the lower right hemithorax, with normal heart beat frequency and without murmurs. The patient was hemodynamically stable with a heart rate of 70 bpm, blood pressure of 146/84 mmHg and afebrile. The remaining physical examination was normal.

**Figure 1:**
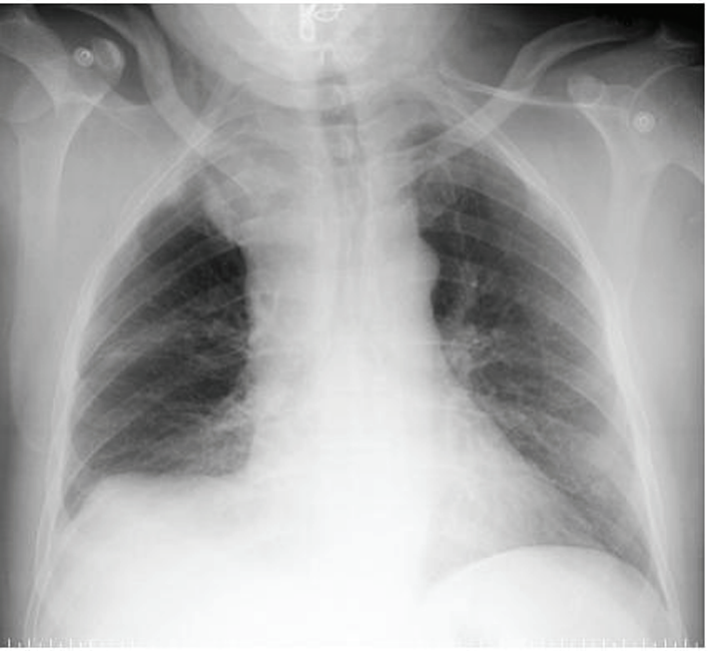
Chest radiograph in posteroanterior projection is shown in which a pleural effusion in the right lower field and a consolidation image at the top ipsilateral chest and adjacent to the mediastinum can be observed.

**Figure 2:**
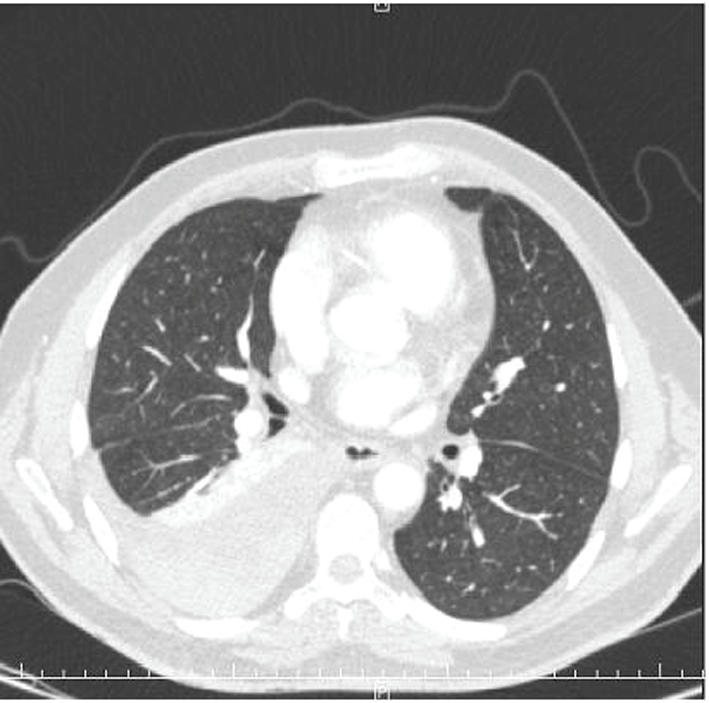
Cervicothoracicangio-CT showing a moderate right pleural effusion with underlying passive atelectasis.

**Figure 3:**
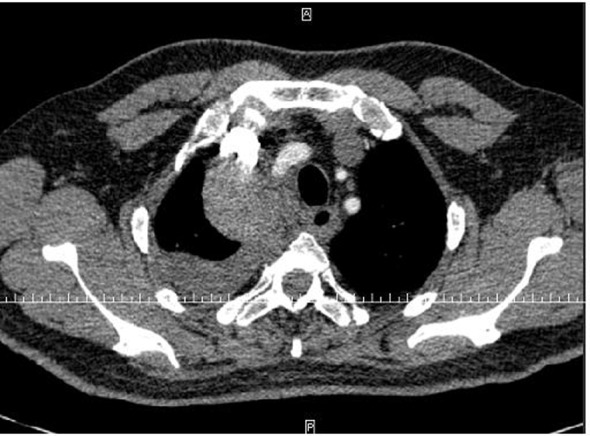
CT angiography showing a loculation in the right upper paramediastinal cervicothoracic region, with active bleeding points dependent on the ascending cervical branch of right subclavian artery.

During his hospital stay, several diagnostic tests were performed. The admission blood analysis reported 14,600 leukocytes, 266,000 platelets and 4,900,000 erythrocytes with hemoglobin of 12.1 g/dL and hematocrit of 37.4%. The rest of the values of the blood count and biochemistry, including liver and kidney functions, were within normal limits. The posteroanterior and lateral chest radiographs showed pleural effusion in the lower right field and an image in the ipsilateral vertex adjacent to the mediastinum (Fig. 1). Given these findings, a cervicothoracic angio-CT (computed tomography) was performed, which showed moderate right pleural effusion with passive atelectasis. These findings and the level of hematocrit, measured in the effusion, were compatible with a diagnosis of hemothorax. In addition, a loculation in the right upper mediastinal was found, with active bleeding points from the cervical branch of the right subclavian artery (Fig. 2, 3).

**Figure 4:**
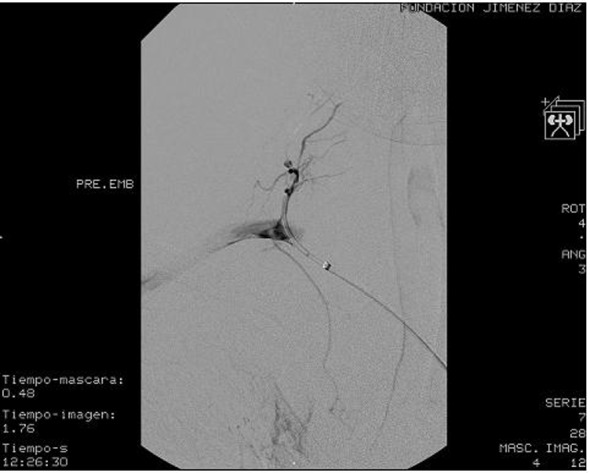
Brachiocephalic arteriography of the right opercular-cervical region where we observe the presence of bleeding dependent of the cervical muscular branch of the right subclavian artery.

**Figure 5:**
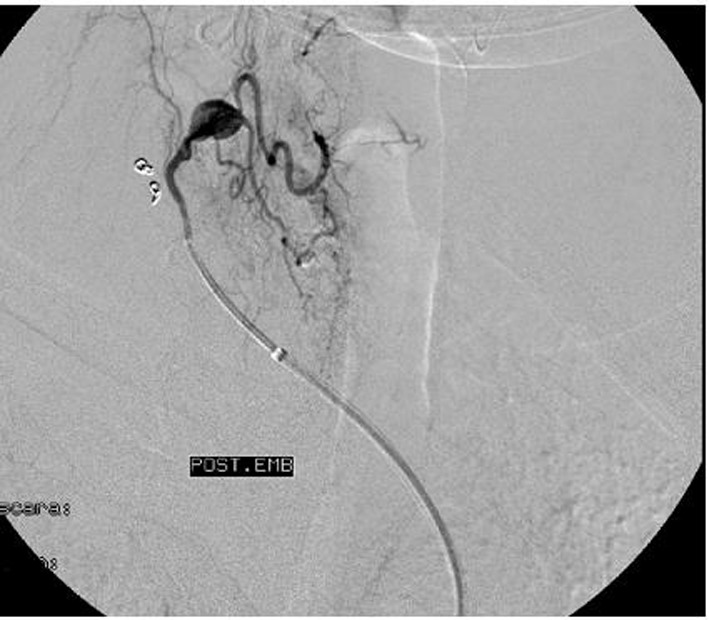
Brachiocephalic arteriography of the right opercular-cervical region where we observe the presence of a pseudoaneurysm of 5 × 5 mm, dependent upon the inferior thyroid branch of the right subclavian artery.

Hemoglobin levels declined during the first 2 days of admission from 12.1 to 9.6 g/dL, although the patient remained hemodynamic stable. A supraaorthic arteriography of the right cervical-opercular region was performed, which showed the presence of bleeding from the cervical muscular branch of the right subclavian artery, which was selectively catheterized and embolized with coils, with satisfactory results (Fig. 4). During this procedure, as an incidental finding, we observed the presence of a pseudoaneurysm of 5 × 5 mm, dependent on the inferior thyroid branch of the right subclavian artery (Fig. 5). After discussing the different therapeutic possibilities, we decided not to treat this lesion, because of the technical complexity and the risks of embolization in that territory, and a conservative approach was finally decided.

Afterwards, a video-assisted thoracoscopy was made, in order to drain the hemothorax and to carry out a visual review of the pleural cavity. We evacuated 800 mL of bloody effusion with clots. There were no signs of active bleeding. Finally, the two thoracic drainages were removed 48 h after surgery, with an output of 190 mL of serosanguineous fluid. The patient had a good radiologic progression, presenting, at hospital discharge, a chest X-ray without infiltrates or pleural effusion and resolution of the loculated image on the right apex. Finally, after a year of follow-up, the patient has remained clinically asymptomatic with no further episodes of active bleeding.

## Discussion

Vascular lesion due to NF-1 has been extensively studied, and some case series suggest an incidence of 3.6% [4]. Arterial lesions affecting both large and small vessels can be found at the abdominal aorta or renal, mesenteric, carotid-vertebral or intracranial vessels. Spontaneous rupture is rare, but some cases have been described in intercostals, popliteal, intracranial and retroperitoneal arteries. Right subclavian artery branches are exceptionally affected [4, 5].

It has been proposed that vascular lesions present in patients with NF-1 occur secondary to two mechanisms related to vessel size. In larger vessels, there is a tendency for aneurysm formation as a side effect of direct invasion of the vascular wall by neurofibromatous tissue or ganglioneuromas, disrupting the integrity of the wall. Invasive tissue generates compression of the vasa vasorum with subsequent ischemia and weakness. Small vessel damage is due to dysplasia of the wall due to proliferation of the intimae and muscularis, loss of the media and fibrosis of the adventitia. This, in turn, initiates a stenosis of the vessel wall, which weakens and causes friability with the subsequent risk of rupture. In addition, small vessel vasculopathy may be related to an abnormality of mesenchymal tissue, and not to neural anomalies [5, 6].

Signs and symptoms vary depending on the size and location of the vascular lesion. Mass effect and vascular ischemic symptoms are the most common. Although the presence of symptoms such as renal hypertension or neurologic symptoms (namely renal artery stenosis, intracranial aneurysm) is common, spontaneous rupture of a large artery is rare [7]. In our case, the presenting symptom was chest pain, which is nonspecific and was probably related to vascular wall damage. The dyspnea that appeared in the second admittance may be related to the pleural effusion demonstrated in the chest radiographs. This sequence of symptoms may explain the clinical timing of the presence of hemothorax in a patient with NF-1. Anatomical diagnosis requires a chest CT or magnetic resonance imaging (MRI) scans [7].Furthermore, performing serial blood tests due to the presence of progressive anemia, as in our patient, could be useful in both diagnosis and prognosis.

Treatment options are dependent on the patient’s hemodynamic stability [5]. Endovascular embolization is indicated if there is hemodynamic stability. This technique requires post-procedural monitoring and a clinical and radiologic assessment, due to the possibility of recanalization, enlarging lesions or collateral vessels [8]. In our patient, coil embolization of the cervical muscle branch of the right subclavian artery was effective in controlling the active bleeding.

As an alternative, thoracotomy with surgical ligation is indicated in cases of active bleeding, with associated hemodynamic compromise. Its main drawback is that the arteries of patients with NF-1 are more friable making surgical control extremely difficult [5]. In this regard, preventive treatment of aneurysms is controversial, especially if the vessels are small because of the technical complexity, thus recommending they be treated conservatively or by coil embolization [9]. In our patient, this complexity and the location of the lesion were the reasons for the conservative treatment of the pseudoaneurysm of the inferior thyroid branch of the right subclavian artery, with good clinical and radiologic results after 1-year follow-up.

Finally, regarding the prognosis of NF-1 patients with a hemothorax, the disease mortality is 36% and postoperative mortality is 33% [5]. In this regard, recently published cases suggest that coil embolization offers the best results [5, 9]. Improvements in diagnosis, a more accurate angio-CT, and treatment with coil embolization can improve the management of the disease, as well as prevent the rupture of vessels and, therefore, improve survival.
